# Safe and effective performance of pediatric spinal deformity surgery in patients unwilling to accept blood transfusion: a clinical study and review of literature

**DOI:** 10.1186/s12891-021-04081-3

**Published:** 2021-02-19

**Authors:** Alexander Mihas, Subaraman Ramchandran, Sebastian Rivera, Ali Mansour, Jahangir Asghar, Harry Shufflebarger, Stephen George

**Affiliations:** 1 Florida International University Herbert Wertheim College of Medicine, 11200 SW 8th Street, Miami, FL 33199 USA; 2 Department of Orthopedic Surgery, Nicklaus Children’s Hospital, 3100 SW 62nd Avenue, Miami, FL 33155 USA; 3 Department of Orthopedic Surgery, Jackson Memorial Hospital, University of Miami, 1611 NW 12th Avenue, Miami, FL 33136 USA; 4 Cantor Spine Institute, 3000 Bayview Drive Suite 200, Fort Lauderdale, FL 33306 USA; 5 Paley Orthopedic and Spine Institute at St. Mary’s Medical Center, 901 45th Street, West Palm Beach, FL 33407 USA

**Keywords:** Scoliosis, Blood loss, Jehovah’s witness, Blood transfusion, Blood salvage, Spinal deformity correction

## Abstract

**Background:**

Pediatric deformity surgery traditionally involves major blood loss. Patients refusing blood transfusion add extra clinical and medicolegal challenges; specifically the Jehovah’s witnesses population. The objective of this study is to review the safety and effectiveness of blood conservation techniques in patients undergoing pediatric spine deformity surgery who refuse blood transfusion.

**Methods:**

After obtaining institutional review board approval, we retrospectively reviewed 20 consecutive patients who underwent spinal deformity surgery and refused blood transfusion at a single institution between 2014 and 2018. We collected pertinent preoperative, intraoperative and most recent clinical and radiological data with latest follow-up (minimum two-year follow-up).

**Results:**

Twenty patients (13 females) with a mean age of 14.1 years were identified. The type of scoliotic deformities were adolescent idiopathic (14), juvenile idiopathic (1), neuromuscular (3) and congenital (2). The major coronal Cobb angle was corrected from 55.4° to 11.2° (80% correction, *p* <  0.001) at the latest follow-up. A mean of 11.4 levels were fused and 5.6 levels of Pontes osteotomies were performed. One patient underwent L1 hemivertebra resection and three patients had fusion to pelvis. Estimated blood loss, percent estimated blood volume loss, and cell saver returned averaged 307.9 mL, 8.5%, and 80 mL, respectively. Average operative time was 214 min. The average drop in hemoglobin after surgery was 2.9 g/dL. The length of hospital stay averaged 5.1 days. There were no intraoperative complications. Three postoperative complications were identified, none related to their refusal of transfusion. One patient had in-hospital respiratory complication, one patient developed a late infection, and one patient developed asymptomatic radiographic distal junctional kyphosis.

**Conclusions:**

Blood conservation techniques allow for safe and effective spine deformity surgery in pediatric patients refusing blood transfusion without major anesthetic or medical complications, when performed by an experienced multidisciplinary team.

**Level of evidence:**

Level IV.

## Background

Surgical intervention for spinal deformities is associated with substantial blood loss [1], which is of special concern in pediatric patients due to their smaller blood volume in comparison to adults [2]. Allogeneic blood transfusion rates in pediatric patients undergoing posterior spinal fusion have been reported to range from 17.5 to 19.3% [3, 4], with some institutions reporting rates as high as 31% [5]. However, allogeneic blood transfusions are associated with various risks: infections, immunomodulation, hemolytic reactions, allergic reactions, circulatory overload, and acute lung injury [6, 7]. Furthermore, one study showed that transfusion of just a single unit of blood prolonged hospital stay length and increased postoperative morbidity in patients who underwent elective spine surgery, independent of patient comorbidities and preoperative hematocrit levels [8].

In addition to the potential complications of allogeneic blood transfusions, patients may refuse blood transfusions due to personal or religious reasons. The Jehovah’s Witnesses (JW) are a Christian denomination who notably refuse blood transfusions due to their interpretation of several biblical passages that refer to blood as sacred and prohibit its consumption [9]. As a result, JW do not accept whole blood (including autologous) nor its main components of plasma, red blood cells, white blood cells, and platelets; however, each member is left to their own personal discretion in regards to accepting blood fractions (e.g. immunoglobulins and clotting factors) [10].

Performing invasive procedures in patients who do not accept blood products poses ethical, clinical, and medicolegal challenges for surgical personnel. Such circumstances create friction between a physician’s duty to provide optimal care and respect patient autonomy [11]. Moreover, caring for these patients requires thorough preoperative screening and management for conditions such as anemia and coagulopathy [12, 13]. From a legal perspective, although competent adults reserve the right to refuse medical treatment for themselves [14], if parental decisions endanger a child’s life, the child’s welfare takes priority [15]. These challenges unfortunately become more complex if the situation involves minors or elective procedures since court decisions have been inconsistent in these cases [15, 16].

Due to the morbidity associated with blood loss and transfusion, and the growing population of patients who do not accept blood products, numerous blood conservation techniques have been developed in an attempt to improve patient outcomes [17, 18]. Preoperative strategies mainly consist of raising hemoglobin levels with recombinant human erythropoietin or iron supplementation [19, 20]. Perioperative techniques include proper patient positioning [21], controlled hypotensive anesthesia (HA) [22–24], acute normovolemic hemodilution (ANH) [25], intraoperative cell salvage (ICS) [26–28], topical hemostatic agents [29], antifibrinolytics (e.g. aminocaproic acid and tranexamic acid) [30–35], and lastly, certain surgical tools can also reduce blood loss such as full electrocautery dissection, bipolar tissue sealants [36], and ultrasonic bone cutting tools for performing osteotomies [37, 38]. It should be noted that JW patients may request that ANH and ICS equipment maintain continuity with their vascular system as they may otherwise equate these methods to autologous blood transfusion [9]. Few studies have investigated the use of blood conservation techniques in spinal deformity procedures in patients who are unwilling to accept blood products [16, 39]. The objective of this study is to evaluate the outcomes of adolescent patients who underwent spinal deformity correction procedures and refused blood transfusions. We hypothesize that by employing various blood conservation techniques, these procedures can be performed safely and effectively by an experienced multidisciplinary team.

## Methods

After obtaining Institutional Review Board approval, a retrospective review of radiographic and medical records was performed to identify patients who refused blood transfusion prior to undergoing spinal deformity surgery between 2014 and 2018 at a single institution. All patients underwent single stage, posterior instrumentation and fusion for scoliosis with the use perioperative blood salvage techniques. Exclusion criteria included any patient that (1) received blood transfusion or blood products, or (2) underwent revision surgery.

All patients received a detailed preoperative hematology work-up and iron supplementation for 4 weeks prior to surgery. Preoperative discussion involved detailed advantages and risks of surgical procedure. All patients and family were questioned about their preferences regarding acceptance of cell saver blood. The families were counselled regarding the possibility of staged procedure. Clinically, an intraoperative blood loss of more than 50% of estimated blood volume or hypotension requiring pressors to maintain the mean arterial pressure of 75 mmHg was used as a threshold for staging the procedure. Intraoperative blood salvage techniques were also used for all patients. Prior to incision, all patients received a bolus of 50 mg/kg of tranexamic acid, followed by continuous infusion of 5 mg/kg. Hypotensive anesthesia was performed during surgical dissection with a mean arterial pressure of 60–70 mmHg. Skin incision was injected with 1% Lidocaine with 1:100,000 epinephrine solution. Careful sub-periosteal dissection was carried out using electrocautery and bipolar tissue sealer device was used as needed to aid with hemostatic control. Furthermore, topical tranexamic acid (TXA) was used in the form of 1:1 dilution with normal saline as TXA soaked sponges, which were packed in areas of wound that were outside of area of interest. Surgifoam (Johnson & Johnson), an absorbable gelatin powder, was used over decorticated surfaces, Ponte osteotomy sites, and pedicle screw holes for additional hemostasis. In addition, for facetectomies and when applicable, vertebral body decancellation was performed using ultrasonic bone cutting devices. Cell saver was used throughout procedure to capture blood loss. Hemoglobin was measured preoperatively and monitored throughout the intraoperative and postoperative periods. Superficial drains were used in all patients, which were removed on the first postoperative day after first mobilization.

Data collected from patient records included patient demographics (age, gender, body mass index, diagnosis and comorbidities); radiographic variables (curve type, coronal Cobb angles, truncal shift, thoracic kyphosis, spinopelvic parameters); surgical variables (estimated blood loss [EBL], percent estimated blood volume loss [% EBVL], operative time, number of fusion levels, type and number of osteotomies, number of thoracoplasty levels, type of instrumentation, iliac fixation and interbody fusion if employed) and perioperative outcomes including complications, length of stay in ICU, hospital length of stay, intraoperative and postoperative hemoglobin. EBL was calculated using the blood collected in the cell saver and weighted soaked sponges after taking into consideration the amount of saline irrigation used and was recorded periodically throughout the procedure. Estimated blood volume (EBV) was calculated as 70 mL/kg (body weight) [1]. Frequency distributions were completed for demographic and perioperative variables. All continuous variables are presented mean ± standard deviation. Preoperative and postoperative radiographic measurements were compared using paired sample t-tests, where *p*-values of < 0.05 were considered significant. Analysis was performed separately for idiopathic and non-idiopathic (congenital and neuromuscular scoliosis) patients. All statistics were conducted with SPSS 25 software.

## Results

### Demographic and surgical variables

A total of 20 patients who refused blood transfusions met the inclusion criteria for this study, 13 (65%) of which were females. The study population had a mean age of 14.1 ± 2.4 years and a mean BMI of 22.8 ± 5.8. Of these patients, 14 patients (70%) had adolescent idiopathic scoliosis (AIS), 1 (5%) had juvenile idiopathic scoliosis (JIS), 2 (10%) had congenital, and 3 (15%) had neuromuscular scoliosis. Among the 14 patients with AIS, Lenke classifications were as follows: 1 (7.1%) type 1A curve, 5 (35.7%) type 1B curves, 1 (7.1%) 1C curve, 1 (7.1%) type 3C curve, 4 (28.6%) type 5C curves, and 2 (14.2%) 6C curves. Mean Risser score for patients in the study was 3.8 ± 1.3. None of the idiopathic or congenital patients had any medical comorbidities. Two of the patients with neuromuscular scoliosis had comorbidities including severe gastroesophageal reflux, seizure disorder and restrictive lung disease. One of the patients also had severe cardiac comorbidities including patent ductus arteriosus (repaired), coarctation of aorta (repaired) and bicuspid aortic valve.

There was a mean of 11.4 ± 3.1 levels fused and 5.6 ± 3.1 levels of Pontes osteotomies, as well as 3.7 ± 1.8 levels of thoracoplasty in the idiopathic group. One patient (5%) received a hemivertebra resection and three patients (15%) received iliac fixation. Means for EBL, % EBVL, cell saver returned, and total fluids transfused were 307.9 ± 134.8 mL, 8.5 ± 5.0%, 80.4 ± 56.0 mL, and 2201.2 ± 562.7 mL, respectively, with a mean operative time of 214.6 ± 65.4 min. None of the patients in the study were staged due to increased intraoperative blood loss. Separate values for idiopathic and non-idiopathic groups can be found in Table 1.

**Table 1:** Demographics, surgical variables, and immediate postoperative outcomes in adolescent patients with spinal deformity correction who refused blood transfusion

	Idiopathic (*n* = 15)	Non-Idiopathic (*n* = 5)
**Demographic Variables**		
Age (years)	14.7 ± 2.6	12.4 ± 0.9
Gender (n, %)		
Female	10 (66.7%)	3 (60%)
Male	5 (33.3%)	2 (40%)
BMI (kg/m^2^)	22.5 ± 5.4	23.9 ± 7.8
Diagnosis (n, %)		
AIS	14 (93.3%)	0
JIS	1 (6.7%)	0
Congenital	0	2 (40%)
Neuromuscular	0	3 (60%)
Hemoglobin (g/dL)	13.3 ± 0.9	13.8 ± 1.5
**Surgical Variables**		
Number of Fusion Levels	10.9 ± 2.3	13.0 ± 4.8
Number of Ponte Osteotomies	5.7 ± 2.5	5.8 ± 5.6
Number of Thoracoplasty Levels	3.7 ± 1.8	0
Hemivertebra Resection (n, %)	0	1 (20%)
Iliac Fixation (n, %)	0	3 (60%)
EBL (mL)	286.3 ± 126.0	372.6 ± 154.2
% EBVL	7.2 ± 3.1	12.7 ± 7.7
Cell Saver Returned (mL)	74.6 ± 56.2	96.6 ± 58.3
Total Fluids Transfused (mL)	2114.7 ± 510.3	2461.0 ± 693.0
Operative Time (min)	197.7 ± 61.5	265.2 ± 53.4
**Postoperative Outcomes**		
Hemoglobin POD 1 (g/dL)	11.5 ± 1.2	10.6 ± 2.2
Hemoglobin POD 2 (g/dL)	10.6 ± 1.1	9.8 ± 2.0
Decrease in Hemoglobin (g/dL)	2.7 ± 0.9	3.9 ± 2.9
Number of Days in ICU	0	1.2 ± 2.2
Length of Stay in Hospital (days)	4.9 ± 0.6	5.4 ± 2.1
In-hospital Complications (n, %)	0	1 (20%)
Late Complications (n, %)	0	2 (40%)

*BMI* Body mass index, *EBL* Estimated blood loss, *AIS* Adolescent idiopathic scoliosis, *JIS* Juvenile idiopathic scoliosis, *% EBVL* Percent estimated blood volume loss, *POD* Postoperative day

### Outcome variables

**Fig. 1:**
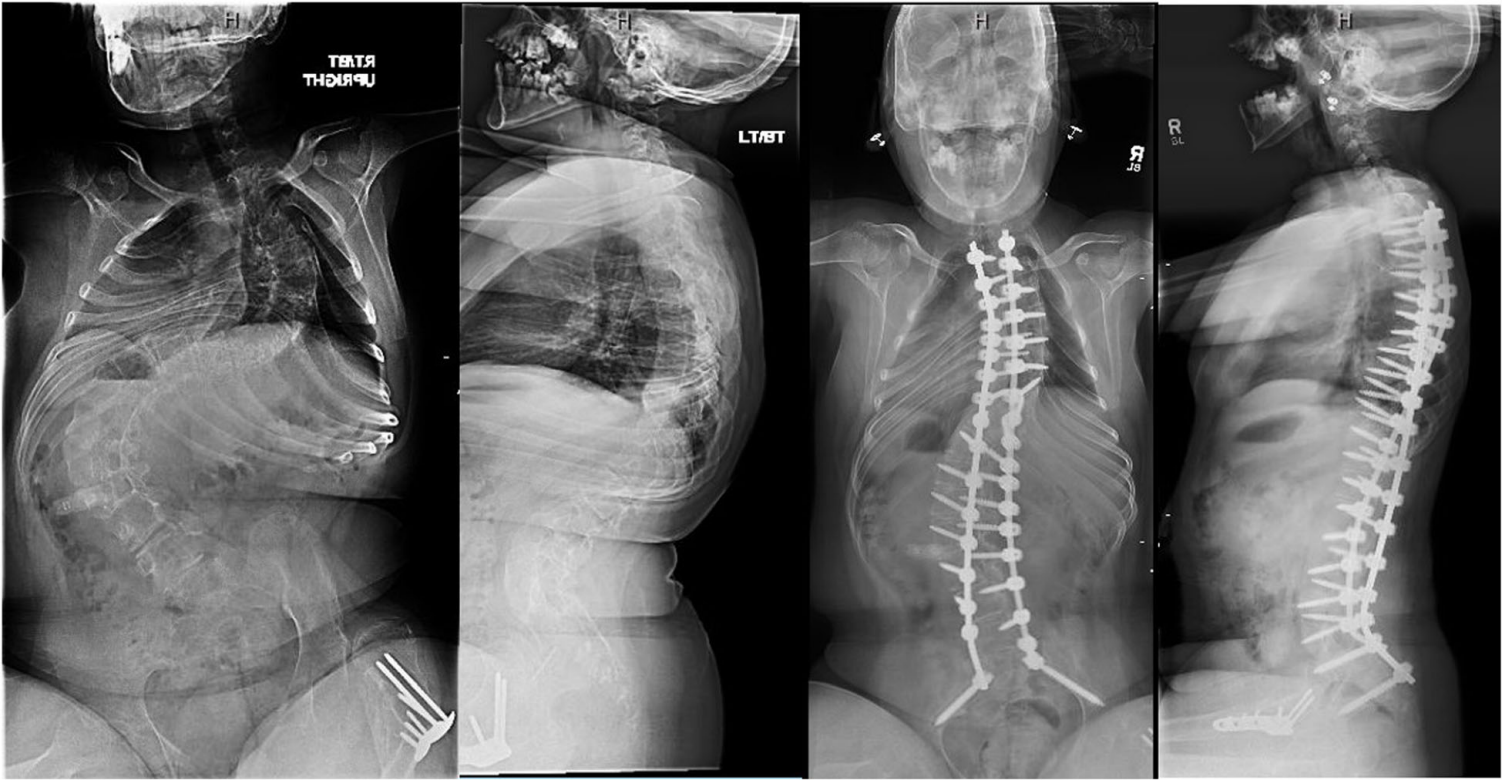
Pre- and 1-year postoperative anteroposterior and lateral radiographs of a 13-year-old female with spastic quadriplegic cerebral palsy treated by posterior spinal fusion from T2 to pelvis showing satisfactory correction. Intraoperative blood loss was 400 mL and perioperative decrease in Hb was 2.3 mg/dL

**Fig. 2:**
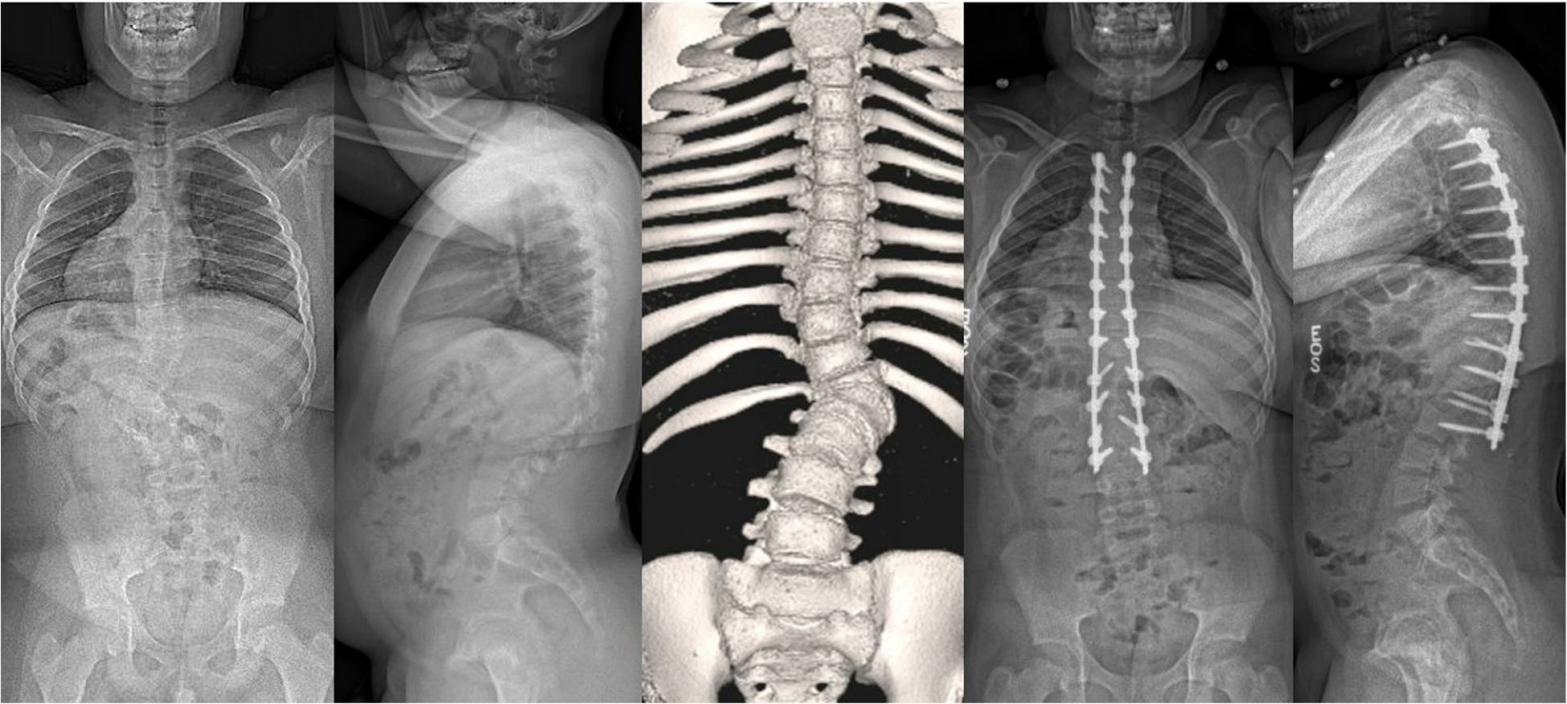
Pre- and 2-year postoperative anteroposterior and lateral radiographs and 3D CT of a 13-year-old male with congenital scoliosis treated by posterior spinal fusion with L1-hemivertebra resection. Intraoperative blood loss was 100 mL. The 2-year postoperative radiographs show presence of radiographic distal junctional kyphosis which remains asymptomatic

The mean decrease between preoperative and postoperative hemoglobin levels was 2.7 ± 0.9 g/dL in the idiopathic group and 3.9 ± 2.9 g/dL in the non-idiopathic group. There were no intraoperative complications and the average length of hospital stay was 4.9 ± 0.6 days and 5.4 ± 2.1 days in the idiopathic and non-idiopathic groups, respectively. Three postoperative complications were identified, which were not related to refusing transfusions. One patient had perioperative atelectasis requiring respiratory support, one patient developed a late infection, which needed antibiotics with revision and one patient developed asymptomatic radiographic distal junctional kyphosis, which also required revision (Table 1). Two of the neuromuscular scoliosis patients were transferred to ICU (5 days and 1 day, respectively) after surgery. At latest follow-up (minimum two-year follow-up), the mean major coronal Cobb angle was corrected from 49.6 ± 6.4° to 7.8 ± 3.1° in the idiopathic group and 72.8 ± 29.4° to 21.0 ± 11.8° in the non-idiopathic group (Fig. 1). The patient who underwent a hemivertebra excision at L1 had a change in the coronal Cobb angle from 43° to 11° with an EBL and % EBVL of 100 mL and 1.7%, respectively (Fig. 2). Perioperative changes in radiographic and clinical variables can be found in Table 2.

**Table 2:** Comparison of preoperative to postoperative radiographic and clinical variables in adolescent patients with spinal deformity correction who refused blood transfusion

	Preoperative	Postoperative	*P* value
**Coronal major Cobb angle**			
Idiopathic	49.6 ± 6.4°	7.8 ± 3.1°	**< 0.001**
Non-Idiopathic	72.8 ± 29.4°	21.0 ± 11.8°	**0.005**
**Thoracic kyphosis**			
Idiopathic	27.8 ± 11.1°	33.6 ± 8.4°	**0.005**
Non-Idiopathic	49.8 ± 18.2°	40.4 ± 7.1°	0.237
**Lumbar lordosis**			
Idiopathic	49.9 ± 10.5°	52.5 ± 8.3°	0.184
Non-Idiopathic	51.2 ± 14.0°	49.2 ± 9.4°	0.733
**Hemoglobin**^a^ **(g/dL)**			
Idiopathic	13.3 ± 0.9	10.6 ± 1.1	**< 0.001**
Non-Idiopathic	13.8 ± 1.5	9.8 ± 2.0	0.169

*P* values in bold represent significance.

^a^Comparison made between preoperative and postoperative day 2 values

## Discussion

Blood loss is considered to be a major cause of morbidity in pediatric spinal deformity surgery. However, blood transfusions involve various risks and may be refused due to personal or religious reasons, most notably by the Jehovah’s Witnesses. Surgical treatment of these patients presents unique challenges for physicians and medical staff, as they require rigorous preoperative evaluation and the combined use of numerous blood conservation techniques. It is thus important to emphasize that the practicability and efficacy of performing such procedures in those who refuse transfusions depends on having an experienced multidisciplinary team, which typically consists of hematologists, surgeons, anesthesiologists, and other surgical personnel. All of our patients in this series underwent standard preoperative and surgical techniques as described in our methods. Blood conservation techniques used included iron supplementation, hypotensive anesthesia, intraoperative cell salvage, tranexamic acid, electrocautery and bipolar tissue sealants, and ultrasonic bone cutting devices. Average blood loss was 307.9 mL, which is lower than the average blood loss for posterior spinal fusion in pediatric scoliosis patients (500 mL–1000 mL) [1]. The average postoperative hemoglobin was 10.5 g/dL, with the lowest being 7.3 g/dL, which occurred in a patient with neuromuscular scoliosis who underwent fusion from T2 to the pelvis. The patients in our series had an average improvement in their major coronal Cobb angle from 55.4° to 11.1° (correction of 80%, *p* < 0.001), with minimal complications. Specifically, average improvement in the major coronal Cobb angle was 49.6 ± 6.4° to 7.8 ± 3.1° (correction of 84%, *p* < 0.001) in the idiopathic group and 72.8 ± 29.4° to 21.0 ± 11.8° (correction of 71%, *p* = 0.005) in the non-idiopathic group (Table 2).

Hypotensive anesthesia (HA) is a well-documented method for reducing blood loss [22–24]. A notable 1992 study by Brodsky et al. showed that HA reduced blood loss in JW patients undergoing scoliosis surgery compared to case-matched controls who received normotensive anesthesia [22]. However, the JW patients who received HA also had shorter operative times, and this study determined that the most important determinant of blood loss is operative technique. Furthermore, Sum et al. demonstrated that using HA in scoliosis surgery decreased average blood loss by 55%, reduced the need for transfusion by 53%, and shortened average operative time by 81 min [40]. Although one concern regarding HA in spine surgery is the risk of spinal cord ischemia, especially when combined with ANH, current literature supports its use [41, 42].

Intraoperative cell salvage (ICS) or cell saver is the process of collecting blood lost during surgery for the purpose of filtering red blood cells for reinfusion. ICS was employed for all of our patients (average reinfusion of 80 mL) since majority of JW patients accept this blood conservation method as long as its equipment remains continuous with their circulation [9]. Several studies have shown ICS reduces the need for transfusion in scoliosis patients undergoing posterior spinal fusion [26–28]. In addition, a 2017 systematic review by Stone et al. claimed that ICS may be cost effective when considering the total costs involved with allogeneic blood transfusions [27]. Cost comparison of ICS and blood transfusions is clearly not a factor in patients who refuse transfusions.

Numerous studies have demonstrated that antifibrinolytics reduce perioperative blood loss and transfusion rates in pediatric patients undergoing scoliosis surgery [30–35]. In a study by Grant et al. on the comparative efficacy of high-dose TXA (20 mg/kg loading, 10 mg/kg/hr. maintenance) vs. low-dose TXA (10 mg/kg loading, 1 mg/kg/hr. maintenance), the authors reported that high-dose TXA showed a trend toward a reduction in transfusion requirements compared to low-dose TXA [43]. Based on the above data, literature from adult populations, and our own clinical experience, we use a loading dose of 50 mg/kg and a maintenance dose of 5 mg/kg/hr. [44]. Analogous to systemic TXA, topical TXA has been used in various surgical specialties to decrease perioperative blood loss. Although current evidence is limited in the spine literature [45, 46], topical TXA used in total joint arthroplasty procedures has shown significant promising results [47, 48]. In a placebo-controlled study, Krohn et al. investigated the impact of topical TXA on postoperative blood loss in patients undergoing elective lumbar spine fusions and found that 2–5 min of locally applied TXA solution before wound closure reduced the postoperative drain output by 50% [49]. At our institute, we use topical TXA in the form of sponges soaked in 1 g TXA in 100 ml of normal saline to be used to pack the wound during different stages of the procedure including the end of exposure, thoracoplasty, and during instrumentation.

The use of ultrasonic bone cutting tools in pediatric spinal deformity surgery is relatively recent, particularly for osteotomies and facetectomies, owing to their tissue selectivity and ability to reduce blood loss [37, 38]. The blades of these tools operate via short excursions at frequencies of over 22,500 oscillations per second. On contact, these repetitive impacts sever rigid structures while soft tissues are much less affected due to their elasticity. Furthermore, these tools reduce bleeding from bone decortication by cauterizing cancellous bone, which is responsible for most of the blood loss in posterior spinal fusion [22]. A study by Bartley et al. was the first to investigate the effects of ultrasonic bone scalpels (UBS) on reducing blood loss in AIS patients undergoing posterior spinal fusion [37]. In the UBS group, UBS was used for facetectomies and apical Ponte-type posterior releases, while standard osteotomes and rongeurs were used in the control groups. The UBS group had 30 to 40% less blood loss, as well as less cell saver transfused and blood loss per level fused, compared to controls. Operative times were identical among all groups. A 2019 study by Wahlquist et al. corroborated these results, including in patients with neuromuscular scoliosis [38].

Our study has several limitations. First, we have a small sample size. Although the relative size of our target population makes it difficult to gather a larger sample size, it is from a single center, which further makes our results less generalizable. Second, we cannot make confident conclusions regarding the ideal blood conservation protocol for this patient population as we lack a control group. Third, multiple blood conservation techniques were used in each patient, so we are unable determine which specific methods are the most efficacious. Lastly, our study was a retrospective review.

## Conclusions

In summary, through the use of iron supplementation, hypotensive anesthesia, intraoperative cell salvage, tranexamic acid, and ultrasonic bone cutting tools, surgical correction of spinal deformities can be safely performed in adolescent patients refusing blood transfusion with excellent deformity correction and minimal complications, by an experienced multidisciplinary team. Our study adds to the scarce body of literature on the use of blood conservation protocols in this patient population, who present ethical, clinical, and medicolegal challenges for healthcare providers. Importantly, such protocols are not only applicable to those who refuse transfusions, but also all patients as their use can minimize blood loss and the need for blood transfusions, which are limited and expensive resources that are associated with various known risks.

## Data Availability

The datasets used and/or analyzed during the current study are available from the corresponding author on reasonable request.

## Abbreviations

BMI: Body mass index
EBL: Estimated blood loss,
% EBVL: Percent estimated blood volume loss
EBV: Estimated blood volume
AIS: Adolescent idiopathic scoliosis
JIS: Juvenile idiopathic scoliosis
UBS: Ultrasonic bone scalpel
JW: Jehovah’s Witness
HA: Hypotensive anesthesia
ANH: Normovolemic hemodilution
ICS: Intraoperative cell salvage
TXA: Tranexamic acid
POD: Postoperative day
